# The association of triglyceride-glucose body roundness index with carotid atherosclerosis in type 2 diabetes: a cross-sectional study

**DOI:** 10.3389/fendo.2026.1952432

**Published:** 2026-10-01

**Authors:** Cuihua Huang, Yan Tong, Yan Luo, Chengwen Xie

**Affiliations:** 1 Longyan First Affiliated Hospital of Fujian Medical University, Longyan, Fujian, China; 2 State Grid Longyan Electric Power Supply Company, Longyan, Fujian, China

**Keywords:** cardiovascular disorders, carotid atherosclerosis, cross-sectional study, triglyceride glucose–body roundness index, type 2 diabetes mellitus

## Abstract

**Background:**

The triglyceride-glucose body roundness index (TyG-BRI) has emerged as a promising biomarker correlated with elevated risks of adverse cardiovascular events. Notably, the potential linkage between TyG-BRI and carotid atherosclerosis (CAS) has not been formally evaluated in individuals with type 2 diabetes mellitus.

**Methods:**

This cross-sectional observational study consecutively enrolled 1060 adults with type 2 diabetes mellitus in Fujian, China. Anthropometric measurements and fasting venous blood biochemical data were collected to calculate TyG-BRI. CAS was ascertained using high-resolution B-mode ultrasound. Spearman correlation analysis was performed to examine the correlations between TyG-BRI and metabolic indicators. Following stepwise adjustment for confounding variables, hierarchical binary logistic regression and restricted cubic spline curves were applied to evaluate the association of TyG-BRI with CAS risk as well as potential effect modifiers. Finally, receiver operating characteristic (ROC) curve analysis was conducted to evaluate the identification capability of TyG-BRI for CAS.

**Results:**

Patients with CAS had significantly higher TyG-BRI levels than non-CAS controls. TyG-BRI was positively correlated with carotid intima-media thickness, glycated hemoglobin (HbA1c), blood pressure, total cholesterol, low-density lipoprotein cholesterol, and uric acid. In fully adjusted models, elevated TyG-BRI was non-linearly associated with higher CAS risk (P for overall association < 0.001, P for non-linearity < 0.01). Per 1-standard-deviation increment in TyG-BRI, the ORs were 1.503 (95% CI 1.281–1.763, P < 0.001) for carotid intima-media thickening, 1.453 (95% CI 1.236–1.709, P < 0.001) for carotid plaque, and 2.247 (95% CI 1.820–2.773, P < 0.001) for CAS. Associations remained consistent across subgroups stratified by age, sex, body mass index (BMI), HbA1c and hypertension, with a stronger effect in participants with BMI < 24 kg/m² (OR 4.838, 95% CI 3.007–7.784, P < 0.001). ROC analysis showed TyG-BRI significantly identified CAS (AUC = 0.698, 95% CI 0.664–0.731, P < 0.001), outperforming TyG and TyG-BMI while matching the performance of the composite indices of TyG combined with waist circumference or waist-to-height ratio.

**Conclusion:**

TyG-BRI demonstrated an independent, non-linear dose-response association with CAS risk in type 2 diabetes. Its integration into cardiovascular risk stratification holds promise for enabling earlier identification of CAS.

## Introduction

Epidemiological data show that there are 589 million adults living with diabetes worldwide in 2024, and the number is projected to rise to 853 million by 2050 (1). As the primary cause of morbidity and mortality in diabetic patients, cardiovascular complications greatly elevate the risks of hospitalization and death. Specifically, patients with type 2 diabetes mellitus face a 2–4-fold higher cardiovascular disease risk than people without diabetes, and cardiovascular events account for approximately 65–75% of mortality among diabetic patients (2, 3). Hence, early detection of cardiovascular diseases and timely targeted prevention are essential to reduce mortality, improve long-term prognosis, and relieve the socioeconomic burden of diabetes.

Atherosclerosis underlies the majority of cardiovascular complications in patients with diabetes, and mounting evidence confirms that insulin resistance and visceral adipose tissue are strongly correlated with atherosclerotic lesions (4–8). The triglyceride-glucose (TyG) index, an inexpensive surrogate biomarker for insulin resistance derived from fasting triglyceride and fasting glucose, is consistently associated with elevated risks of arteriosclerosis, coronary artery disease, and major adverse cardiovascular events (9–12). Complementarily, the recently developed body roundness index (BRI) has been shown to enable precise quantification of visceral adiposity using only simple anthropometric metrics (13). Visceral adipose dysfunction is confirmed to trigger lipid metabolism disorder and inflammation, two pathological drivers that are shown to act synergistically in the process of atherogenesis (14–17). Given this mechanistic background, integrating TyG with BRI yields incremental discriminatory capability for identifying carotid atherosclerosis (CAS). However, the direct association between the TyG-BRI and CAS has not been systematically explored to date.

Against this backdrop, this study investigates the independent association of TyG-BRI with prevalent CAS among Chinese adults with type 2 diabetes mellitus. It further compares its discriminative ability with the standalone TyG index and other TyG-derived anthropometric composites to verify whether TyG-BRI yields superior discriminative ability for CAS.

## Materials and methods

### Selection of study participants

This observational study consecutively enrolled adult inpatients with type 2 diabetes mellitus admitted to the metabolic management center at Longyan First Affiliated Hospital of Fujian Medical University from October 2023 to November 2025. Eligible participants satisfied the following inclusion criteria: (1) age ≥ 18 years; (2) diagnosed with type 2 diabetes mellitus. Individuals were excluded for any of the following: (1) acute diabetic complications including diabetic ketoacidosis or hyperosmolar hyperglycemic state; (2) substantial missing core analysis data; (3) pre-existing severe decompensated cardiovascular, respiratory, hepatic, renal, active infectious or uncontrolled psychiatric diseases; (4) coexisting endocrine diseases (e.g., cushing’s syndrome, pheochromocytoma or hypothyroidism); (5) ongoing use of glucose-lipid homeostasis interfering agents, especially systemic glucocorticoids. The final analysis included 1060 screened participants.

### Collection of general information and laboratory data

Demographic characteristics, clinical parameters and laboratory data were collected by experienced physicians using standardized questionnaires, alongside medical record review and extraction of laboratory results. All investigators received unified training to ensure consistent data collection and minimize measurement bias. Recorded variables encompassed age, sex, diabetes duration, waist circumference (WC), height, body weight, systolic blood pressure (SBP), diastolic blood pressure (DBP), smoking history, alcohol consumption history, medication history, glycated hemoglobin (HbA1c), fasting plasma glucose (FPG), total cholesterol (TC), low-density lipoprotein cholesterol (LDL), high-density lipoprotein cholesterol (HDL), triglycerides (TG), uric acid (UA), serum creatinine (SCr), and alanine aminotransferase (ALT). All participants underwent venous blood sampling after an overnight fast of no less than 8 hours on the morning of testing. Blood specimens were processed and tested immediately following collection under standardized laboratory operating procedures. Biochemical indices were determined on an automatic biochemical analyzer (Roche Diagnostics), while HbA1c levels were measured via high-performance liquid chromatography (Bio-Rad).

Calculation of derived indicators: (1) triglyceride-glucose index (TyG) = ln[TG (mg/dL) × FPG (mg/dL)/2]; (2) body mass index (BMI) = weight (kg)/height² (m²); (3) waist-to-height ratio (WHtR) = WC (cm)/height (cm); (4) BRI = 364.2−365.5 × (1-[WC (cm)/2π]2/[0.5 × height(cm)]2)½; (5) TyG-BMI = TyG × BMI.; (6) TyG-WC = TyG × WC; (7) TyG-WHtR = TyG × WHtR; (8) TyG-BRI = TyG × BRI.

### Detection of CAS

Carotid vascular imaging assessment in this study was performed using a high-resolution two-dimensional B-mode ultrasound system (Philips EPIQ 5) equipped with an L12–5 MHz high-frequency linear array transducer for image acquisition. All participant examinations were performed in strict adherence to standardized positioning protocols. Subjects were placed in the standard supine position with the neck fully exposed and the head slightly extended forward to minimize measurement bias induced by vascular traction. The measurement site for carotid intima-media thickness (cIMT) was uniformly set at the segment 1 cm proximal to the carotid bifurcation. Data were collected from both the left and right carotid arteries, and the arithmetic mean of bilateral measurements was calculated as the final cIMT value (unit: mm). According to the widely accepted diagnostic criteria: carotid intimal thickening (CMT) was defined as cIMT ≥ 1.0 mm; a carotid plaque was identified if either of the following criteria was met: focal wall protrusion exceeding 50% of the thickness of the adjacent normal vessel wall, or focal cIMT ≥ 1.5 mm. In this study, CAS was diagnosed based on the detection of either CMT or carotid plaques. All carotid ultrasound examinations were performed by two technicians possessing more than 5 years of professional experience in vascular ultrasound, and both operators remained strictly blinded to the clinical profiles and laboratory test results. The intra-observer intraclass correlation coefficient (ICC) was 0.94, while the inter-observer ICC was 0.92, confirming the excellent reliability of the carotid ultrasound measurements in this study.

### Statistical analysis

All data entry and statistical analysis were executed in SPSS 24.0. Variables were described as mean ± standard deviation (normal distribution), median (IQR) (skewed distribution), or n (%) (categorical data). Between-group comparisons used appropriate tests: independent t-test and one-way analysis of variance for normal continuous variables, Mann-Whitney U and Kruskal-Wallis tests for non-normal variables, and Chi-square or Fisher’s exact test for categorical data. Spearman correlation was used to examine links between TyG-BRI and cIMT, HbA1c, blood pressure, lipids, and uric acid. Confounder-adjusted logistic regression and restricted cubic spline (RCS) analysis were used to evaluate the relationship between TyG-BRI and CAS risk, followed by hierarchical logistic regression analysis to identify potential effect modifiers. The optimal number of knots for the RCS model was selected based on the minimum Akaike information criterion, with three knots placed at the 10th, 50th, and 90th percentiles of the TyG-BRI distribution. The median TyG-BRI was specified as the reference value, with the corresponding odds ratio set to 1.0. Receiver operating characteristic (ROC) curves were constructed to assess the discriminative power of TyG and its derivative indices for CAS, with the area under the curve (AUC) computed as a summary measure of predictive accuracy. The DeLong test was applied for formal pairwise comparison of AUC estimates across indices. All statistical tests were two-tailed, with a prespecified significance threshold set at α = 0.05, where P < 0.05 was considered statistically significant.

## Results

### Baseline demographic and clinical characteristics of the study participants

As shown in **Figure 1**, a total of 1060 individuals diagnosed with type 2 diabetes mellitus after excluding 85 patients based on exclusion criteria. These participants were subsequently divided into the non-CAS group (n=351) and the CAS group (n=709), with their baseline characteristics summarized in **Table 1**. The overall mean age of the study cohort was 61.05 ± 12.04 years, and males accounted for 55.00% of the total sample. Compared with patients in the non-CAS group, those in the CAS group were significantly older, had a longer duration of diabetes, and exhibited a markedly higher cIMT (P<0.05). Notably, patients in the CAS group presented more severe metabolic disturbances, as evidenced by significantly elevated levels of WC, weight, BMI, HbAlc, SBP, TC, LDL, UA, and SCr (P<0.05). In contrast, HDL were significantly lower in the CAS group (P<0.05). As hypothesized, the CAS group had significantly higher TyG index and all composite TyG-based indices (TyG-BMI, TyG-WC, TyG-WHtR, TyG-BRI) (P<0.05).

**Figure 1:**
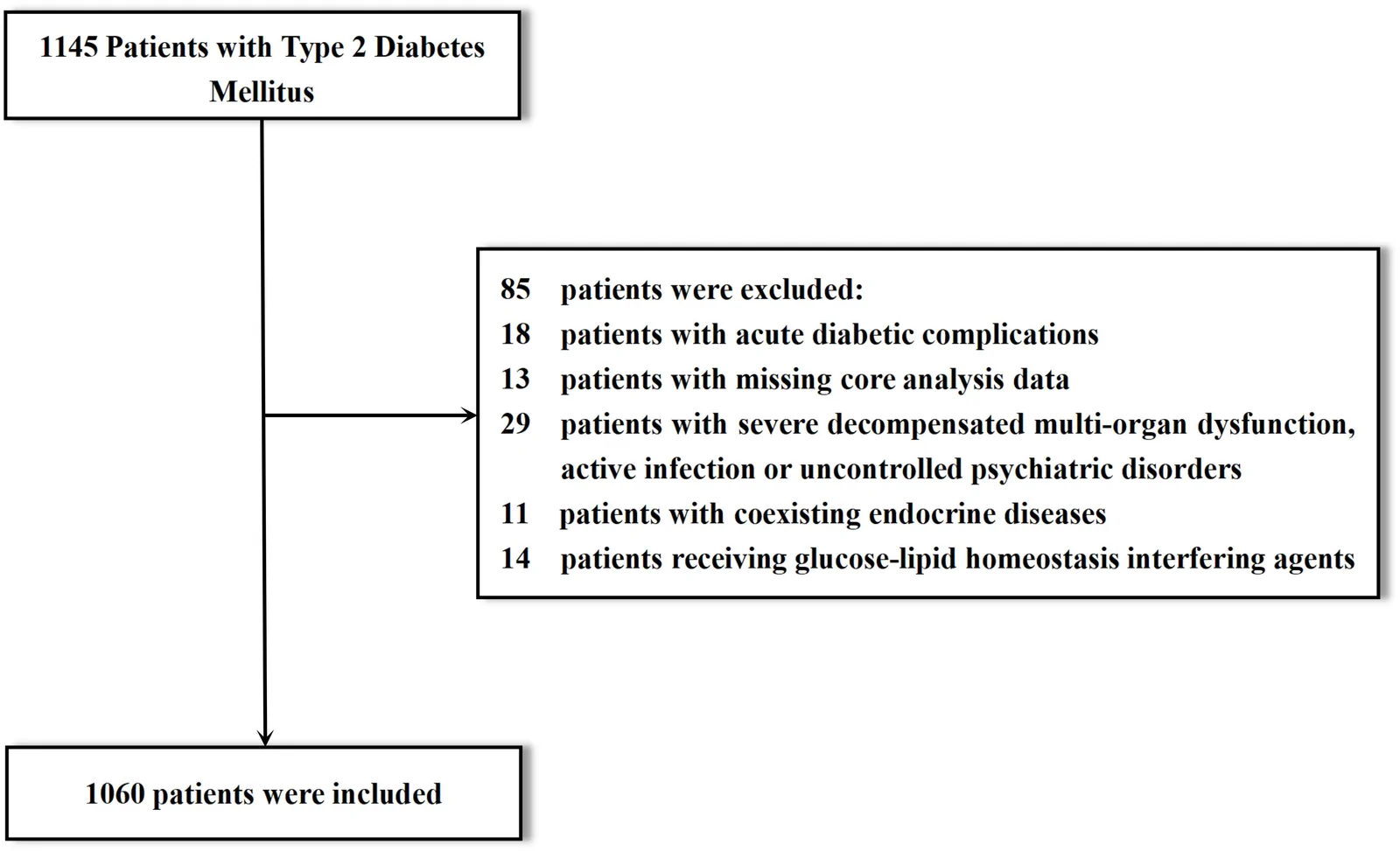
Participant screening flow chart.

**Table 1:** Baseline characteristics of CAS and non-CAS groups.

Variable	Total (n=1060)	Non-CAS (n=351)	CAS (n=709)	P value
Age (years)	61.05 ± 12.04	53.62 ± 11.67	64.74 ± 10.43	<0.001
Male, n(%)	583 (55.00%)	186 (52.99%)	397 (55.99%)	0.390
Diabetic duration (year)	9.12 ± 7.09	7.17 ± 6.64	10.08 ± 7.11	<0.001
cIMT (mm)	0.92 ± 0.22	0.75 ± 0.11	1.01 ± 0.21	<0.001
WC (cm)	89.40 ± 8.68	85.47 ± 8.79	91.34 ± 7.94	<0.001
Height (cm)	160.67 ± 8.30	161.89 ± 8.23	160.07 ± 8.28	0.001
Weight (kg)	64.09 ± 10.96	62.59 ± 11.37	64.83 ± 10.69	0.002
BMI (kg/m^2^)	24.77 ± 3.43	23.80 ± 3.31	25.25 ± 3.39	<0.001
HbAlc (%)	9.03 ± 2.21	8.84 ± 2.21	9.12 ± 2.20	0.047
FPG (mmol/L)	6.85 ± 2.47	6.66 ± 2.26	6.95 ± 2.56	0.069
SBP (mmHg)	138.53 ± 20.81	131.01 ± 18.74	142.25 ± 20.79	<0.001
DBP (mmHg)	78.91 ± 11.49	79.38 ± 11.63	78.68 ± 11.42	0.353
TC (mmol/L)	4.62 ± 1.14	4.49 ± 1.06	4.69 ± 1.18	0.007
LDL (mmol/L)	2.91 ± 0.96	2.78 ± 0.85	2.98 ± 1.01	0.001
HDL (mmol/L)	1.08 ± 0.30	1.13 ± 0.33	1.05 ± 0.28	<0.001
TG (mmol/L)	1.83 ± 1.36	1.71 ± 1.37	1.88 ± 1.34	0.050
UA (µmol/L)	329.52 ± 97.10	309.33 ± 85.87	339.52 ± 100.77	<0.001
SCr (µmol/L)	69.07 ± 38.44	61.60 ± 29.84	72.77 ± 41.58	<0.001
ALT (IU/L)	21.00 [15.00, 30.00]	22.00 [15.00, 32.00]	21.00 [15.00, 29.00]	0.213
TyG	8.97 ± 0.70	8.84 ± 0.77	9.03 ± 0.66	<0.001
TyG-BMI	222.75 ± 38.97	211.26 ± 39.57	228.43 ± 37.41	<0.001
TyG-WC	803.42 ± 111.84	758.09 ± 118.22	825.86 ± 101.37	<0.001
TyG-WHtR	5.01 ± 0.71	4.68 ± 0.70	5.17 ± 0.66	<0.001
TyG-BRI	41.11 ± 12.03	35.30 ± 10.46	43.99 ± 11.72	<0.001
Oral hypoglycemic drug, n (%)	867 (81.79%)	277 (78.92%)	590 (83.22%)	0.105
Insulin, n (%)	586 (55.28%)	179 (51.00%)	407 (57.40%)	0.056
Smoking, n (%)	296 (27.92%)	85 (24.22%)	211 (29.76%)	0.069
Drinking, n (%)	113 (10.66%)	30 (8.55%)	83 (11.71%)	0.143
Hypertention, n (%)	568 (53.58%)	112 (31.91%)	456 (64.32%)	<0.001
Statins, n (%)	110 (10.38%)	24 (6.84%)	86(12.13%)	0.008
Antiplatelet agents, n (%)	81 (7.64%)	14 (3.58%)	67(9.45%)	0.002

CAS, carotid atherosclerosis; cIMT, carotid intima-media thickness; WC, waist circumference; BMI, body mass index; HbA1c, hemoglobin A1c; FPG, fasting plasma glucose; SBP/DBP, systolic/diastolic blood pressure; TC, total cholesterol; LDL/HDL, low-/high-density lipoprotein cholesterol; TG, triglycerides; UA, uric acid; SCr, serum creatinine; ALT, alanine aminotransferase; TyG, triglyceride-glucose Index; WHtR, waist to height ratio; BRI, body roundness index; Statins (represented by atorvastatin and rosuvastatin); Antiplatelet agents (represented by aspirin and clopidogrel).

### Carotid artery lesions demonstrated significantly elevated TyG-BRI levels

TyG-BRI levels were significantly increased in patients with CMT, carotid plaque, or CAS, suggesting that these adverse carotid-related outcomes were likely associated with higher TyG-BRI (as shown in **Figure 2**, P <0.05).

**Figure 2:**
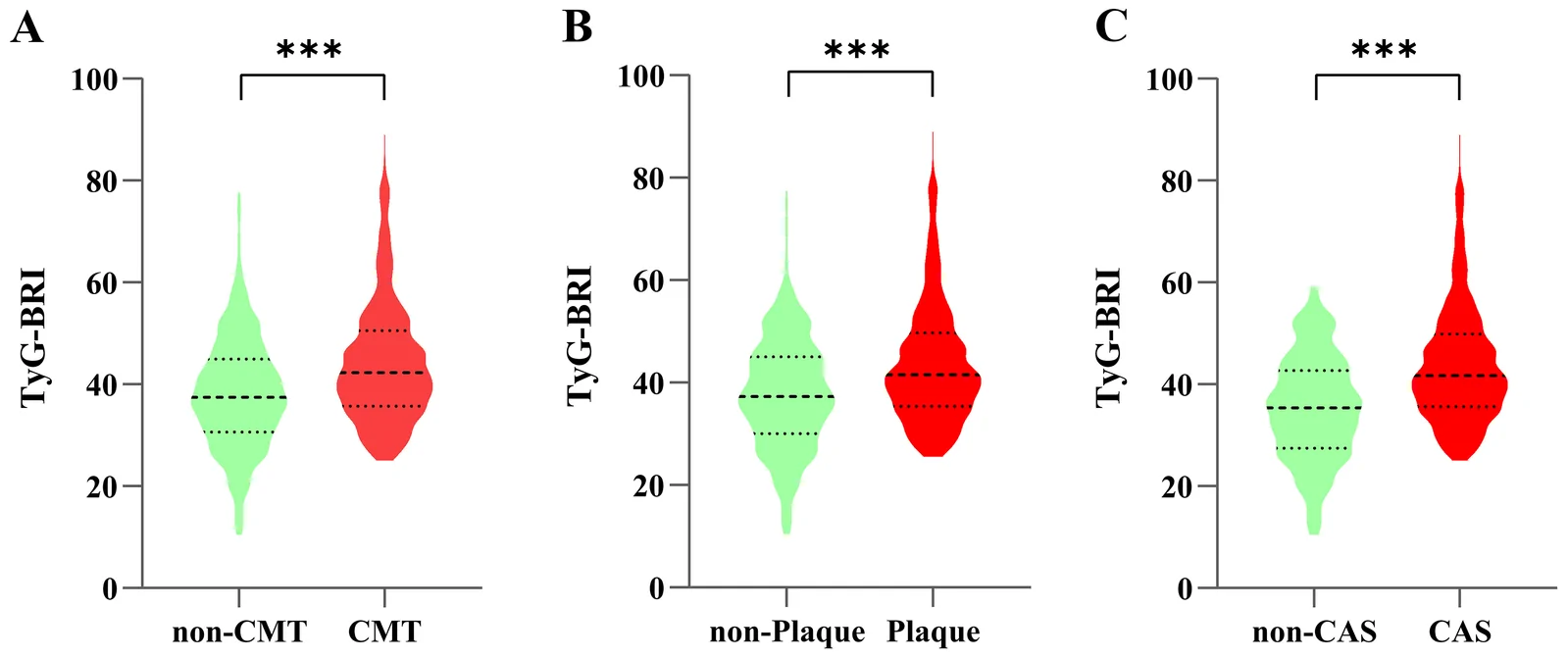
TyG-BRI levels in patients with carotid artery lesions. TyG, triglyceride-glucose Index; BRI, body roundness index; CMT, carotid intima-media thickening; CAS, carotid atherosclerosis. *** P value <0.001.

### Correlations of TyG-BRI with cIMT and metabolic profiles

As visualized in the correlation heatmap presented in **Figure 3**, TyG-BRI demonstrated a statistically significant positive correlation with cIMT (r = 0.224, P < 0.05). Further correlation analysis revealed that TyG-BRI was positively associated with multiple conventional cardiovascular risk factors, namely HbA1c, SBP, DBP, TC, LDL and UA, while it was inversely correlated with the vascular protective factor HDL (P < 0.05).

**Figure 3:**
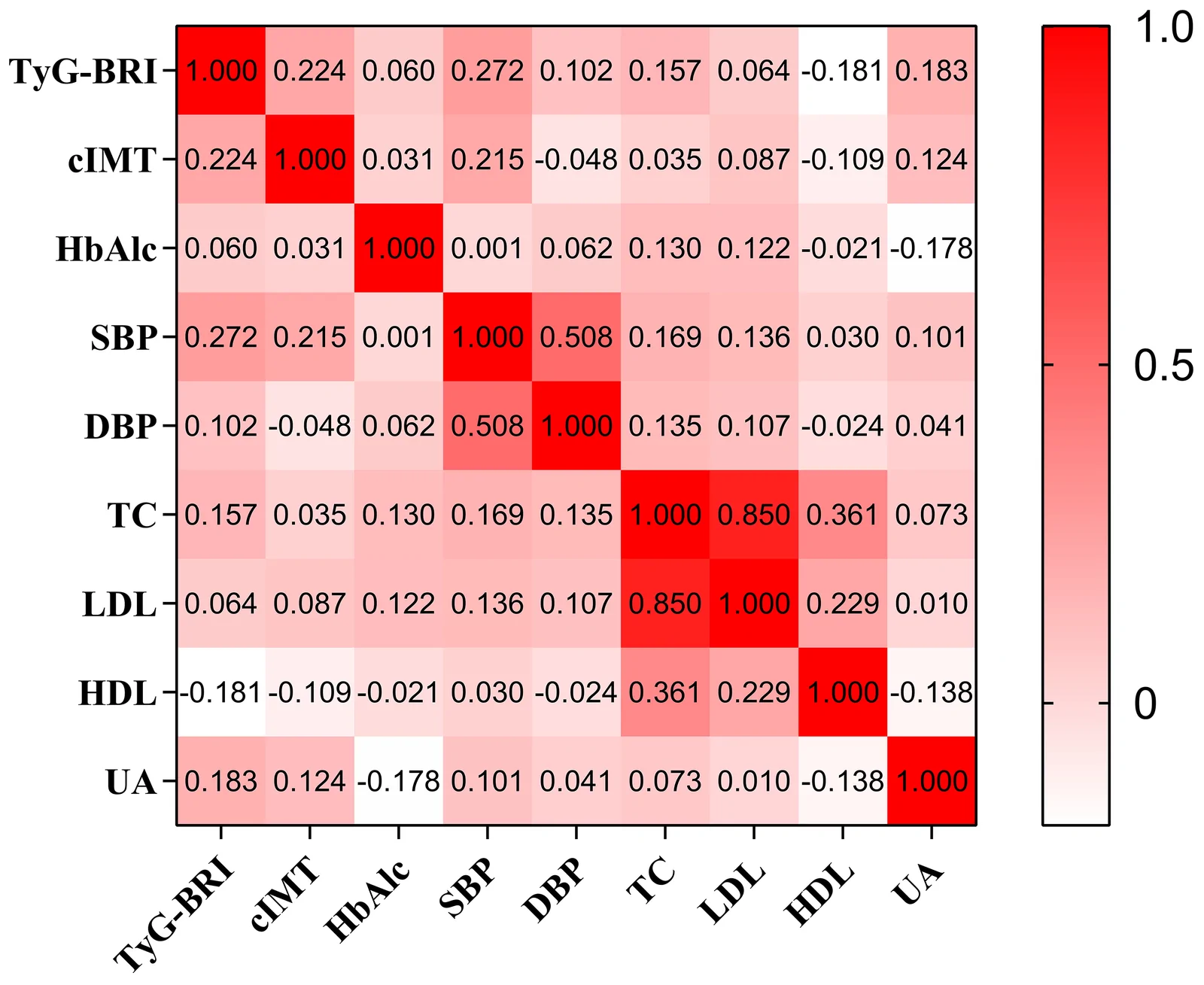
Correlation analysis heatmap. TyG, triglyceride-glucose Index; BRI, body roundness index; cIMT, carotid intima-media thickness; HbA1c, hemoglobin A1c; SBP/DBP, systolic/diastolic blood pressure; TC, total cholesterol; LDL/HDL, low-/high-density lipoprotein cholesterol; UA, uric acid.

### TyG-BRI exhibited a significant independent non-linear relationship with carotid artery lesions

Following multivariable adjustment for potential confounders, the elevated TyG-BRI level was significantly correlated with carotid artery lesions, as visualized in **Figure 4**. In detail, per 1-standard-deviation increment in TyG-BRI, independent associations were observed for CMT (OR: 1.503, 95% CI: 1.281–1.763, P < 0.001), carotid plaque (OR: 1.453, 95% CI: 1.236–1.709, P < 0.001), and CAS (OR: 2.247, 95% CI: 1.820–2.773, P < 0.001). Further RCS analysis confirmed that all these dose-response relationships were statistically significant non-linear associations (P for overall association < 0.001, P for non-linearity < 0.01).

**Figure 4:**
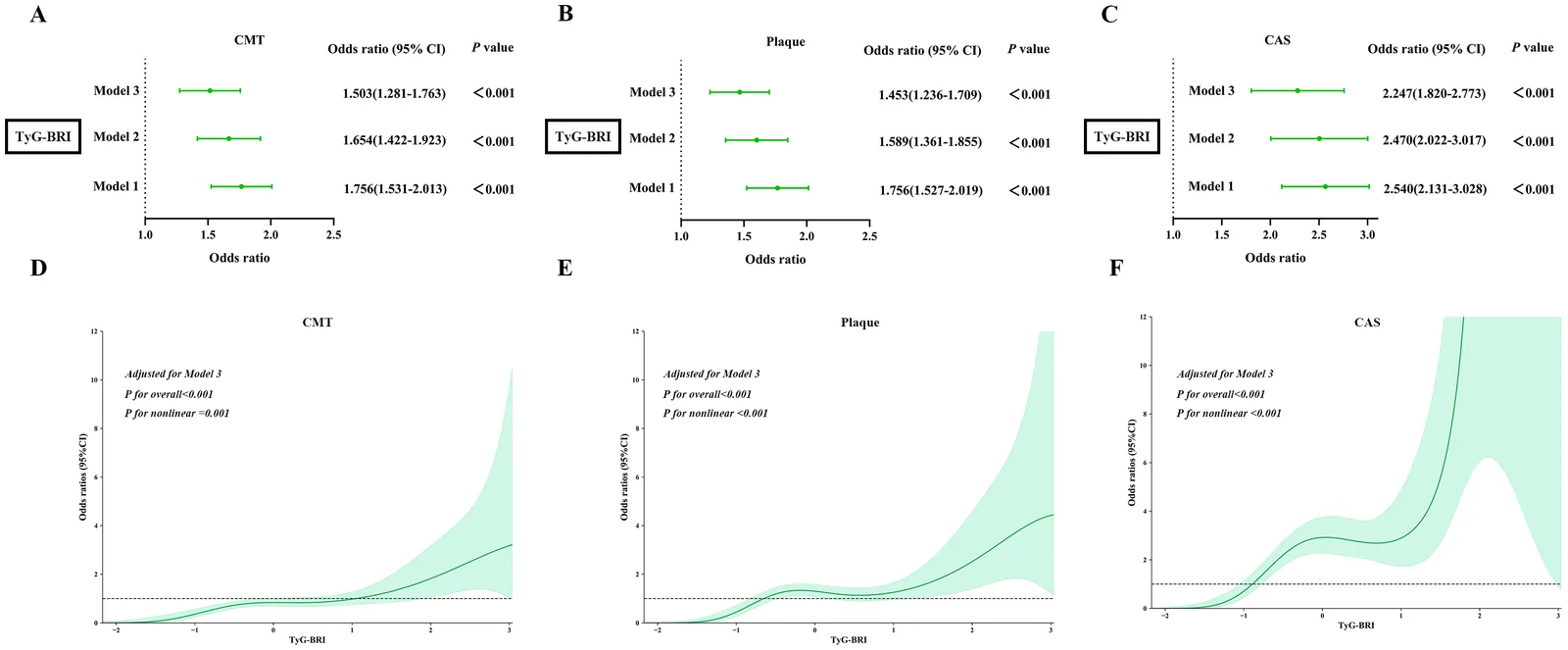
Logistic regression analysis and RCS curves of TyG-BRI with CMT, carotid plaque and CAS. **(A–C)** Logistic regression analysis; **(D–F)** RCS curves. Model 1: Unadjusted. Model 2: adjusted for sex, age and duration of diabetes mellitus. Model 3: further adjusted for hemoglobin A1c, systolic blood pressure, diastolic blood pressure, total cholesterol, low-density lipoprotein cholesterol, high-density lipoprotein cholesterol, uric acid, creatinine, alanine aminotransferase, oral hypoglycemic drug, iusulin, smoking, statins.

### Stratified logistic regression analysis of TyG-BRI and CAS

Following stratification by sex, age, BMI, HbA1c, and hypertension, TyG-BRI remained independently correlated with CAS (P < 0.001), as shown in **Figure 5**. This relationship was significantly stronger among participants with BMI < 24 kg/m² (P for interaction < 0.001), with no evidence of effect modification by age, sex, HbA1c, or hypertension status (P for interaction > 0.05).

**Figure 5:**
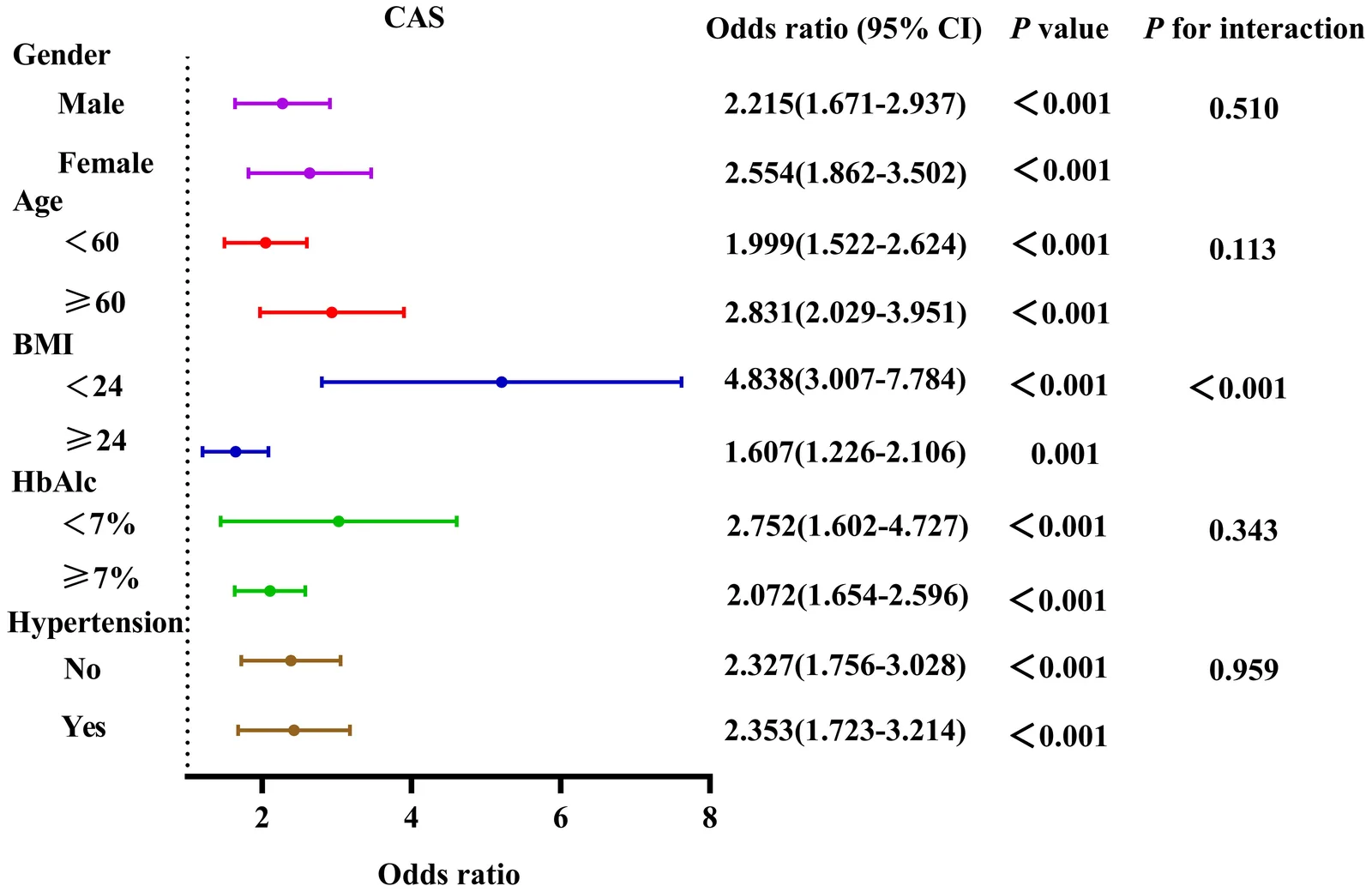
Stratified logistic regression analysis was performed to investigate the association between TyG-BRI and CAS, with sex, age, BMI, HbA1c, and comorbid hypertension as stratifying variables. TyG, triglyceride-glucose Index; BRI, body roundness index; CAS, carotid atherosclerosis; BMI, body mass index.

### Identification capability of TyG-BRI for CAS

As shown in **Figure 6** and **Table 2**, the discriminative ability of TyG-BRI for CAS was evaluated using ROC analysis. TyG-BRI presented an AUC of 0.698 (95% CI: 0.664–0.731, P < 0.001), with a sensitivity of 68.7% and specificity of 60.1% at the optimal cut-off point. Notably, the AUC of TyG-BRI was significantly higher than those of TyG and TyG-BMI (P < 0.05), and its identification capability was comparable to that of TyG-WC and TyG-WHtR (P > 0.05).

**Figure 6:**
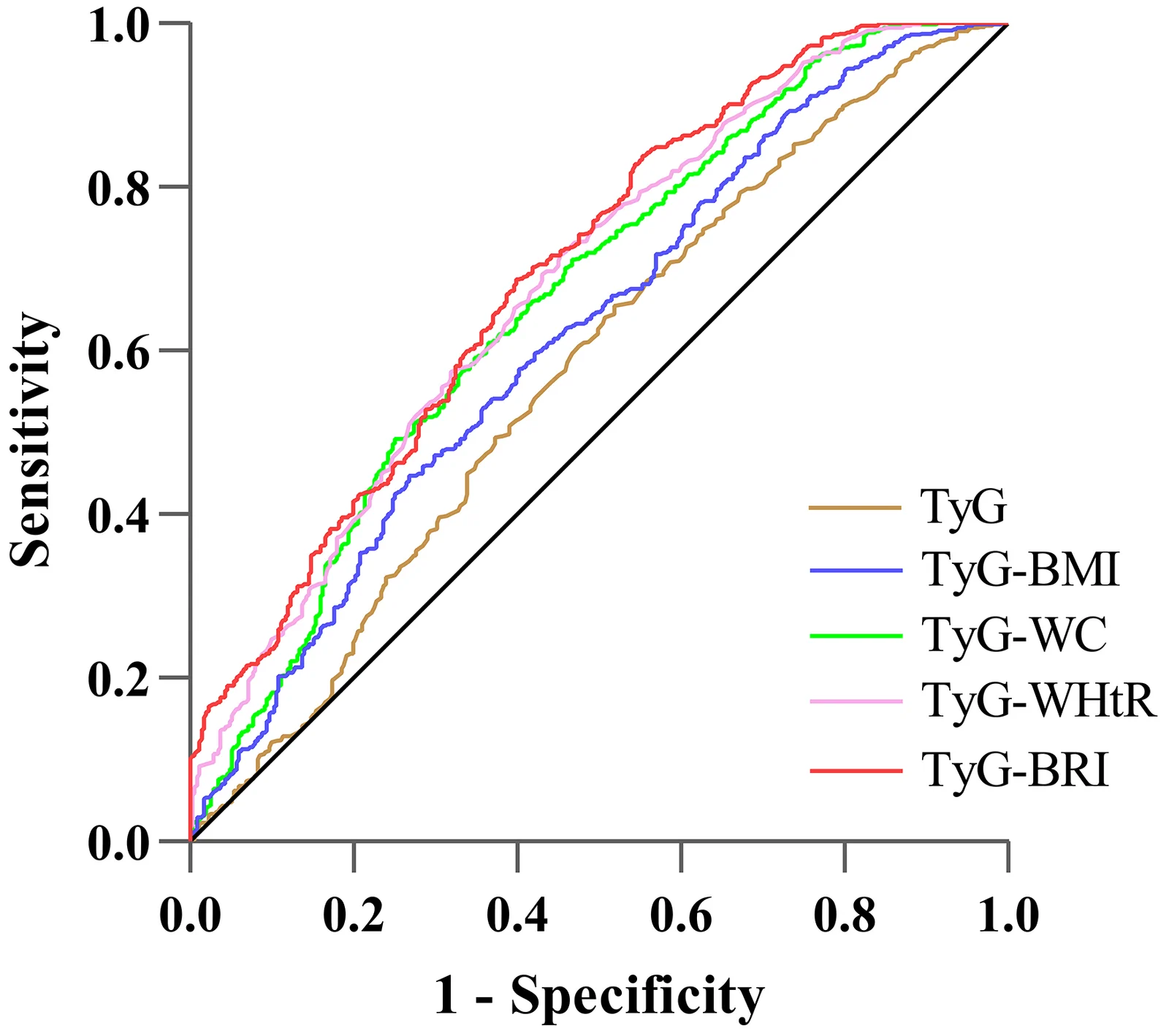
ROC analysis for identifying CAS using TyG and its composite indices. CAS, carotid atherosclerosis; TyG, triglyceride-glucose Index; BMI, body mass index; WC, waist circumference; WHtR, waist to height ratio; BRI, body roundness index.

**Table 2:** ROC analysis for CAS identification using TyG and its composite indices.

Variables	Sensitivity (%)	Specificity (%)	AUC (95% CI)	P value(vs TyG-BRI)
TyG	0.654	0.481	0.577 (0.540-0.615)	<0.001
TyG-BMI	0.447	0.732	0.621 (0.584-0.657)	0.002
TyG-WC	0.621	0.624	0.665 (0.629-0.701)	0.192
TyG-WHtR	0.721	0.547	0.682 (0.648-0.717)	0.528
TyG-BRI	0.687	0.601	0.698 (0.664-0.731)	/

TyG, triglyceride-glucose Index; BMI, body mass index; WC, waist circumference; WHtR, waist to height ratio; BRI, body roundness index.

## Discussion

Type 2 diabetes mellitus populations bear disproportionately high cardiovascular morbidity and mortality, with CAS serving as a key preclinical lesion preceding stroke and myocardial infarction. The present study explored the association between the TyG-BRI and CAS, aiming to provide evidence for early vascular risk screening. Collectively, the present results demonstrated that TyG-BRI levels were significantly higher in patients with CAS than in those without carotid lesions, and correlation analysis verified that TyG-BRI was positively correlated with atherosclerotic risk indicators (HbA1c, SBP, DBP, TC, LDL, UA). After adjustment for confounding factors, elevated TyG-BRI exerted an independent and non-linear correlation with CMT, carotid plaque formation and overall CAS burden. Further subgroup analysis showed this association was stronger in patients with BMI < 24 kg/m², while no significant heterogeneity was observed across subgroups stratified by age, sex, HbA1c, or hypertension status. ROC analysis revealed that TyG-BRI yielded higher discriminative ability for CAS than the standalone TyG index and TyG-BMI (AUC, 0.698 vs. 0.577–0.621, all P < 0.05), and no significant difference in discriminative power was detected among TyG-BRI, TyG-WC, and TyG-WhtR.

Accumulating clinical evidence has firmly established that insulin resistance and abdominal obesity represent core pathogenic risk factors for the initiation and progressive aggravation of carotid vascular remodeling and atherosclerosis (16, 18–21). Both pathological states exacerbate macrovascular injury through a cascade of dysmetabolic events. Emerging clinical data demonstrate that insulin resistance concurrently elevates multiple atherogenic risk factors, including fasting plasma glucose, triglycerides, and low-density lipoprotein cholesterol, thus forming a well-documented pathological cascade of “insulin resistance–multiple metabolic disturbances–CAS” (5). A retrospective cohort study enrolling 3,688 Chinese participants confirmed that abdominal obesity, quantified by the visceral adiposity index, is significantly and positively correlated with CAS, and remains a sustained independent association with carotid plaque formation after adjustment for confounders including age and baseline lipid profiles (22). Consistent with these epidemiological observations, interventions targeting the amelioration of abdominal obesity and insulin resistance hold considerable promise for attenuating cIMT and reducing the risk of adverse macrovascular events (23, 24). The synergistic metabolic damage induced by the coexistence of insulin resistance and abdominal obesity provides a core theoretical rationale for the clinical application of composite metabolic indicators, with the TyG-BRI index serving as a representative biomarker.

In recent years, abundant prospective cohort studies and meta-analysis have solidly validated TyG-BRI as a comprehensive metabolic biomarker incorporating glucose homeostasis, lipid profiles and abdominal adiposity, which is closely associated with adverse long-term cardiovascular endpoints in the general population (25–27). A 10-year cohort investigation targeting middle-aged and older adults revealed that individuals in the highest TyG-BRI quartile have a 1.13-fold higher risk of cardiovascular events relative to those in the lowest quartile (28). Further analysis indicated that TyG-BRI exhibits superior discriminative power over the standalone TyG index for cardiovascular episodes, thereby supporting its utility as an independent prognostic marker for adverse cardiovascular outcomes. Consistently, a meta-analysis enrolling more than 3 million participants corroborated that elevated TyG-derived composite metabolic indices markedly elevate the risks of cardiovascular disease, stroke, cardiovascular mortality and all-cause death (29). Beyond general cardiovascular risks, a longitudinal study with a median 13 years of follow-up additionally documented that elevated TyG-BRI correlates with an increased likelihood of cardiovascular-metabolic multimorbidity among hypertensive patients (30). Nevertheless, existing research still has clear limitations. Most published studies concentrate on the general middle-aged and elderly population and hypertensive groups, while direct clinical evidence about the relationship between TyG-BRI and vascular complications in people with type 2 diabetes remains insufficient.

Targeted association analysis were performed in the present study to address the aforementioned research gap. After full adjustment for confounders, we identified a robust independent nonlinear association between TyG-BRI and both CMT and plaque burden. Subgroup analysis showed this association was significantly stronger in participants with normal body weight (BMI < 24 kg/m²). Consistent with Zhu et al., who previously reported that elevated TyG-WHtR preferentially increases cardiovascular-metabolic multimorbidity risk in normal-weight populations, we attribute this pattern to minimal interference from systemic metabolic disorders in this subgroup, where carotid atherosclerotic lesions are primarily driven by isolated insulin resistance, ectopic visceral fat accumulation, subsequent lipotoxicity and chronic inflammation (31). Biomarker performance comparisons further demonstrated that conventional TyG-BMI only reflects systemic obesity status but fails to capture pathological visceral adipose tissue accumulation, limiting its identification capability for CAS in type 2 diabetes. In contrast, novel composite indicators including TyG-BRI, TyG-WC and TyG-WHtR specifically target abdominal adipose metabolic dysfunction (32). These biomarkers effectively capture visceral fat-derived lipotoxic substances and inflammatory mediators.

The pathophysiological link between TyG-BRI and CAS in this population likely involves multiple mechanisms. Inflammatory biomarkers are independently linked to subclinical atherosclerosis in high-risk populations (33). Elevated BRI values indicate excessive visceral fat accumulation, which promotes robust secretion of free fatty acids and pro-inflammatory cytokines—including interleukin-6 and monocyte chemoattractant protein-1—while suppressing the expression of adiponectin, a protective adipokine. This metabolic disturbance induces vascular pathological damage and increases susceptibility to progressive vascular injury (34, 35). Additionally, elevated TyG levels reflect severe insulin resistance, which further aggravates systemic metabolic disorders and triggers excessive platelet activation and endothelial dysfunction (36). Compensatory hyperinsulinemia secondary to insulin resistance aberrantly activates the mitogen-activated protein kinase signaling pathway (37). Chronic exposure to persistent hyperglycemic and hyperlipidemic microenvironments synergistically induces oxidative stress, impairs the endothelial PI3K/Akt/eNOS signaling axis, and reduces nitric oxide production (38–40). Recent studies in populations with metabolic disturbances support the potential complementary value of circulating molecular biomarkers for early cardiovascular involvement (41). Collectively, these pathological alterations may disrupt the carotid endothelial barrier, enhance vascular permeability, and facilitate substantial subintimal deposition of oxidized LDL. Such cascading pathological events ultimately accelerate the initiation and progression of CAS, thereby elevating the long-term risk of adverse cerebrovascular events.

In conclusion, this study confirms that TyG-BRI acts as an independent biomarker for CAS among patients with type 2 diabetes mellitus. TyG-BRI exerts markedly higher discriminatory efficacy for identifying CAS than TyG and TyG-BMI, while exhibiting diagnostic value equivalent to that of TyG-WC and TyG-WhtR. These findings demonstrate that abdominal visceral adiposity, rather than simple generalized obesity, exerts a synergistic effect with insulin resistance in its association with CAS, providing novel insights into the underlying pathophysiological mechanisms of diabetic vascular injury.

Several limitations should be noted in this study. First, the cross-sectional design cannot establish a causal relationship between elevated TyG-BRI and the occurrence of CAS. Second, data regarding the use of SGLT2 inhibitors and GLP-1 receptor agonists, which exert well-documented effects on body weight, metabolic parameters and cardiovascular protection, were not collected in this study. Furthermore, unmeasured lifestyle variables and missing medication dosage data may introduce residual confounding bias. Third, this single-center study recruited hospitalized patients with suboptimal glycemic control and a high burden of carotid artery disease. These factors restrict the generalizability of our findings to community-dwelling populations and limit the utility of the TyG-BRI index for population screening.

## Data Availability

The raw data supporting the conclusions of this article will be made available by the authors, without undue reservation.
